# The Cyclic Peptide Cyclo-zp80r Controls *Salmonella enterica* and *Listeria monocytogenes* Replication in Non-Concentrated (NFC) Orange Juice: Antibacterial Effects and Mechanisms of Action

**DOI:** 10.3390/foods14142506

**Published:** 2025-07-17

**Authors:** Zhouxia Wang, Ping Zeng, Jinhui Lu, Sharon Shui Yee Leung, Lanhua Yi

**Affiliations:** 1 College of Food Science, Southwest University, Chongqing 400715, China13997833912@163.com (J.L.); 2 College of Biological and Chemical Engineering, Qilu Institute of Technology, Jinan 250200, China; 3 School of Pharmacy, Faculty of Medicine, The Chinese University of Hong Kong, Shatin, Hong Kong 999077, China; sharon.leung@cuhk.edu.hk

**Keywords:** NFC orange juice, food bio-preservative, cyclic peptide, antimicrobial activity, action mechanism

## Abstract

The market for non-concentrated (NFC) orange juice is increasing rapidly due to consumer demand for nutrients and flavor. However, it encounters challenges in microbial safety, particularly from *Salmonella enterica* and *Listeria monocytogenes.* This study aimed to exploit a bio-preservative for NFC orange juice. Results showed that the cyclic peptide cyclo-zp80r had good antibacterial activity, with minimum inhibitory concentration values of 2–8 μM against *S. enterica* and *L. monocytogenes*. It exhibited bactericidal action against *S. enterica* and bacteriostatic action against *L. monocytogenes* at a concentration of 128 μM. This study explored the effect of cyclo-zp80r on the pathogenicity of *S. enterica* and *L. monocytogenes*. The mortality rate of *Galleria mellonella* exposed to these pathogens in NFC orange juice decreased from 100% to 60% after cyclo-zp80r treatment, surpassing the effectiveness of nisin. Cyclo-zp80r exhibited depolarization effects on *S. enterica* and *L. monocytogenes*. It increased outer membrane permeability and damaged the membrane structure of *S. enterica*. Cyclo-zp80r also caused distinct morphological changes, mainly cell collapse in *S. enterica* and localized bubble-like protrusions in *L. monocytogenes*. It induced reactive oxygen species production and DNA binding. The species diversity and abundance in NFC orange juice were also reduced by cyclo-zp80r, particularly in the genera *Pantoea*, *Aeromonas*, *Pseudomonas*, and *Erwinia*. Additionally, cyclo-zp80r exhibited excellent stability at high temperature (121 °C, 5 min) and in fresh orange juice. These results suggest that cyclo-zp80r could be developed as an effective food bio-preservative.

## 1. Introduction

Citrus is a type of Rutaceae fruit that is rich in vitamin C, flavonoids, polyphenols, and other beneficial bioactive compounds [1]. It is known for its unique fragrance, with a sweet and sour taste, making it deeply loved by consumers and extensively planted globally. Its annual production exceeds 150 million tons, making it the third-largest traded agricultural product [2,3,4]. Citrus is one of the most important economic crops in the world; however, the postharvest preservation of citrus is extremely challenging. Losses due to diseases and metabolic disorders affect 30% to 50% of the total yield. Fruit decay caused by fungal infection is considered the most important problem [5,6]. To address these issues, 40–50% of citrus is processed into citrus juice, jam, and canned products to reduce economic losses in the citrus industry [2,4]. Citrus juice, the most widely produced and consumed form, can be classified into fresh juice, concentrated juice (FC), non-concentrated juice (NFC), and orange drinks based on processing methods [7]. Fresh juice is pressed without heat treatment to preserve its original flavor and nutrition. NFC juice undergoes pressing and pasteurization, which preserves the flavor and nutrition while reducing microorganisms. With the advancement of society and the economy, people’s demands for food have evolved from mere satiety to a focus on health and nutrition. Under this trend, NFC orange juice has experienced rapid growth in the market and is increasingly favored by more consumers [8,9]. NFC orange juice is made from fresh orange juice and subjected to pasteurization, high-pressure processing (HPP), pulsed electric field (PEF), or cold plasma treatment [8,10,11]. The heat-sensitive substances (such as aroma and bioactive substances) in concentrated orange juice subjected to traditional high-temperature heat treatment are destroyed [12,13], leading to a decline in sensory quality and nutritional value. In contrast, NFC orange juice better maintains the flavor, color, and nutrients of fresh orange juice, such as vitamin C, flavonoids, anthocyanins, folic acid, mineral K, and other beneficial substances [12,14,15,16]. However, NFC orange juice has problems and challenges in terms of shelf life, stability, and cost [9]. The low-temperature pasteurization method used to sterilize it has limited effectiveness. Orange juice is susceptible to contamination by filamentous *fungi*, *yeasts*, *Bacillus*, *Escherichia coli*, *S. enterica*, and *L. monocytogenes* [10,17,18,19]. These microorganisms lead to a decline in the quality and safety of orange juice, or even render it inedible. *S. enterica* contamination on citrus peels is high and can transfer to the juice during pressing. *S. enterica* can survive in orange juice due to its ability to withstand low pH levels [20]. To maintain its ‘natural and fresh’ qualities, NFC orange juice relies on refrigeration and a cold chain sales approach. However, the inherent cold tolerance of *L. monocytogenes* allows it to grow under cold chain conditions [21]. *S. enterica* and *L. monocytogenes* pose a particular threat to NFC orange juice. To preserve the ‘natural’ characteristics of NFC orange juice and improve its food safety, antimicrobial compounds are needed.

Antimicrobial peptides (AMPs) are small peptides, typically under 100 amino acid residues [22]. They are found widely in plants, animals, microorganisms, and other natural organisms. Natamycin and nisin, the most widely used food bio-preservatives, are AMPs. Natamycin is effective only against fungi, while nisin is effective only against Gram-positive bacteria. Broad-spectrum AMPs, effective against both Gram-positive and Gram-negative bacteria, need to be developed. In our previous study, an antimicrobial peptide, zp80, which can control *L. monocytogenes* in food, was obtained [23]. It is a linear cationic amphiphilic peptide with a typical α-helix structure. To improve its stability, the linear peptide zp80 was modified and cyclized to the circular peptide cyclo-zp80r [24]. However, the application potential of cyclo-zp80r in controlling *S. enterica* and *L. monocytogenes* in NFC orange juice is unknown. The purpose of this study is to investigate the antibacterial activity of cyclo-zp80r against *S. enterica* and *L. monocytogenes*, especially in NFC orange juice, and then to elucidate the antibacterial mechanism of cyclo-zp80r.

## 2. Materials and Methods

### 2.1. Materials

The orange juice used in this study is NFC orange juice produced in Chongqing at 90 °C for 30 s. The antimicrobial peptide cyclo-zp80r was synthesized as in our previous study [24]. Briefly, the L-Arg residue in zp80 was replaced with D-Arg and conjugated with a KKIKK macrolactam ring to obtain cyclo-zp80r, which was purified to over 95% purity using HPLC. Agar powder was purchased from Solarbio in China. LB broth was purchased from Oxoid. TSB broth and MH broth were purchased from Becton, Dickinson and Company. XLT4 agar and PALCAM agar were purchased from Hopebio in China. *Salmonella* strains were isolated from foods and preserved in our laboratory at −80 °C. *L. monocytogenes* ATCC19115 was purchased from the American Type Culture Collection (Manassas, VA, USA) and preserved in our laboratory at −80 °C.

### 2.2. Determination of Antibacterial Activity

Determination of minimum inhibitory concentration (MIC) is an accurate and conventional method for studying the antibacterial activity of antimicrobial peptides [25]. In this experiment, the method proposed by Lü et al. [26] was followed to determine the MIC using the 96-well plate broth dilution method. Briefly, cyclo-zp80r was dissolved in sterile water to prepare a 2 mM stock solution. Then, the stock solution was diluted with MH broth [27] or TSB broth [28] in a 96-well plate to final concentrations of 64, 32, 16, 8, 4, 2, 1, 0.5 μM. Subsequently, 5 μL of *Salmonella* or *L. monocytogenes* suspension with an OD_600 nm_ of 0.08–0.1 was added. The plates were cultured in an incubator at 37 °C for 16–24 h. Each experiment was conducted in triplicate. The MIC value is the minimum concentration of cyclo-zp80r at which no pathogen growth occurs.

### 2.3. Growth Curve and Time–Kill Curve

The effect of cyclo-zp80r on the growth and survival of *S. enterica* and *L. monocytogenes* was evaluated as outlined in our previous methods [29]. For growth curve determination, *S. enterica* was inoculated in fresh LB broth at 2% (*v*/*v*) and cultured in a shaker at 37 °C. The OD_600 nm_ value was measured every hour for 12 h. Once cultured to the logarithmic phase (around OD_600 nm_ = 0.2), cyclo-zp80r was added to a final concentration of 128 μM. For time–kill curve determination, *S. enterica* was cultured to the logarithmic phase in the same way, and cyclo-zp80r was added to a final concentration of 128 μM. The cell suspension was sampled at specific timepoints (0, 2, 4, 6, and 8 h) and then spread on LB plates. Colonies were counted after culturing at 37 °C. *L. monocytogenes* was treated the same as *S. enterica*, except that TSB medium was used instead of LB medium. In addition, sterile water was used as a negative control, and 128 μM nisin was used as a bio-preservative control. Three replications were performed for all groups.

### 2.4. Controlling S. enterica and L. monocytogenes in NFC Orange Juice

The controlling effectiveness of cyclo-zp80r in NFC was evaluated using the method proposed by Sun et al. [30], with some modifications. *S. enterica* and *L. monocytogenes* were cultured to logarithmic phase in LB broth and TSB broth, respectively. Cells were collected by centrifugation and then suspended in sterile saline. The cell suspension was inoculated into NFC orange juice at a concentration of approximately 10^5^ to 10^6^ CFU/mL. Subsequently, cyclo-zp80r was added to the NFC orange juice at a final concentration of 128 μM and stored at 4 °C. The samples were taken out every day for 7 days and spread on XLT4 plates for *S. enterica* counting and PALCAM plates for *L. monocytogenes* counting. Sterile water was used as a negative control. A 128 μM concentration of nisin was used as a bio-preservative control. Each group had three replicates.

To further investigate the controlling effectiveness of cyclo-zp80r, the pathogenicity of *S. enterica* and *L. monocytogenes* in NFC orange juice was evaluated. On the seventh day, the treated NFC orange juice (containing *S. enterica* and *L. monocytogenes*) was injected into *G. mellonella* at a dose of 10 μL of juice per insect. The live/dead ratio of *G. mellonella* was observed every 12 h for 48 h, and the survival rate was recorded. Each group contained 15 *G. mellonella* larvae.

### 2.5. Microbiome

NFC orange juice was divided into two groups. One group was combined with 128 μM cyclo-zp80r, and the other group was combined with sterile water as a control. Each treatment was conducted in triplicate. After 7 days of storage at 4 °C, each group underwent microbiome analysis at Novogene Bioinformatics Co., Ltd. (Beijing, China) based on 16S rRNA amplicon sequencing. The cetyltrimethylammonium bromide (CTAB) method was used to extract DNA from the samples, followed by PCR amplification on a gradient PCR instrument (BIO-RAD, Hercules, CA, USA). A universal DNA purification kit (TIANGEN, Beijing, China) was used for DNA recovery and purification. Finally, Sequencing was performed using a NovaSeq 6000 sequencer (Illumina, San Diego, CA, USA). The obtained data were subjected to abundance and diversity analyses.

### 2.6. Stability

The thermal stability and resistance of cyclo-zp80r to enzymes in fresh orange juice were investigated by measuring the MIC. The cyclo-zp80r stock solution was treated at 121 °C for 5 min, 65 °C for 30 min, or 37 °C in fresh orange juice for 2 h, and then MIC was determined using the 96-well plate broth dilution method. To assay for resistance to enzymes in fresh orange juice, cyclo-zp80r was added to the fresh orange juice to a final concentration of 1 mM and treated at 37 °C for 2 h. *S. enterica* was used as the indicator.

### 2.7. Membrane Potential

The membrane potential was analyzed according to our previous study [31], with some modifications. *S. enterica* and *L. monocytogenes* were cultured to the logarithmic phase and then centrifuged and resuspended in sterile saline to OD_600 nm_ = 1.88–1.95. Sterile KCl (100 mM) was added, followed by 1 μM fluorescent probe DiSC_3_(5) (Sigma, St. Louis, MO, USA). After incubating in the dark for 15 min, cyclo-zp80r was immediately added to final concentrations of 1 μM and 4 μM. Fluorescence intensity was measured at an excitation wavelength of 620 nm and an emission wavelength of 670 nm on a microplate reader (Synergy H1, BioTek, Shoreline, WA, USA). Sterile water was used as the negative control, and 1 μg/mL of valinomycin was used as the positive control. The experiments were conducted in triplicate in each group.

### 2.8. Determination of Outer Membrane’s Permeability

The permeability of the outer membrane of *S. enterica* was evaluated according to the method described by Meng et al. [32], with slight modification. *S. enterica* was cultured to the logarithmic phase. After washing with sterile saline, cells were suspended to OD_600 nm_ = 0.4. The NPN fluorescent probe was added at a final concentration of 10 μM. Subsequently, 32 μM and 128 μM cyclo-zp80r were then immediately added. The fluorescence intensity was measured using a microplate reader (Synergy H1, BioTek, USA) at an excitation wavelength of 350 nm and an emission wavelength of 429 nm after incubation in the dark for 15 min. Sterile water was used as the negative control, and 4 μg/mL melittin was used as the positive control. Each experiment was conducted in triplicate.

### 2.9. Transmission Electron Microscopy (TEM)

Following the method described by Yi et al. [33], *S. enterica* was cultured in LB medium to the logarithmic phase and then treated with 32 μM and 128 μM of cyclo-zp80r for 2 h. Cells were fixed with 2.5% glutaraldehyde overnight at 4 °C, washed, and then fixed with osmic acid for 2 h. Treated cells were dehydrated on a gradient with 10%, 30%, 50%, 70%, 80%, 90%, and 100% ethanol. Subsequently, cells were infiltrated with 25%, 50%, 75%, and 100% white resin, and then embedded in white resin by drying. Finally, a 100 nm ultrathin section was prepared using an EM UC7 Ultramicrotome (Leica, Wetzlar, Germany) and then covered with a copper mesh. Samples were double-stained with uranium acetate and lead citrate. The ultrastructure of cells was observed on a transmission electron microscope (HT7800, Hitachi, Tokyo, Japan).

### 2.10. Scanning Electron Microscopy (SEM)

SEM was conducted following the method described by Mentor et al. [34], with some modifications. *S. enterica* and *L. monocytogenes* were cultured to the logarithmic phase (OD_600 nm_ = 0.2) and then treated with 32 μM and 128 μM of cyclo-zp80r for 2 h. The cells were resuspended in sterile saline, dropped onto a coverslip to dry naturally, and then fixed in 2.5% glutaraldehyde overnight at 4°C. Gradient ethanol dehydration was performed in the same way as for the TEM. After drying, the cells were coated with gold. The cells were observed using a scanning electron microscope (Regulus8230, Hitachi, Japan).

### 2.11. Determination of Intracellular Reactive Oxygen Species (ROS)

ROS were measured as described in our previous study [29], with some modifications. *S. enterica* and *L. monocytogenes* cultured to the logarithmic phase were resuspended in sterile saline to OD_600 nm_ = 0.6. The cell suspension was divided into two groups, and 20 mM L-ascorbic acid was added to one of the groups. Subsequently, 2 μM, 4 μM, 8 μM, 16 μM, 32 μM, 64 μM, and 128 μM cyclo-zp80r were added to each group and cultured at 37 °C for 2 h. Finally, 10 μM 2′,7′-dichlorofluorescein diacetate (DCFH-DA) was added. The fluorescence intensity was measured at an excitation wavelength of 488 nm and an emission wavelength of 525 nm on a microplate reader (Synergy H1, BioTek, USA) after 30 min of incubation in the dark.

### 2.12. DNA–Cyclo-zp80r Binding Gel Retardation Assay

Gel retardation assay was conducted according to the method described by Wang et al. [35], with slight modification. Firstly, genomic DNA was extracted from *S. enterica* and *L. monocytogenes* according to the instructions provided with the bacterial genomic DNA extraction kit (Accurate Biology, Guangzhou, China). A total of 10 μL DNA (296 μg/mL) was mixed with an equal volume of different concentrations of cyclo-zp80r solution to final concentrations of 0, 18.5, 37, 74, 148 μg/mL (the same concentration of DNA in each group). The samples were then incubated at 37 °C for 2 h. Electrophoresis was performed on a 0.8% agarose gel, then the samples were observed and photographed using a gel imaging system (OI 2000, Bio-oi, Zhenjiang, China).

### 2.13. Statistical Analysis

Each experiment was performed in triplicate, and the results were expressed as mean ± standard deviation. One-way analysis of variance and Duncan test were performed using IBM SPSS Statistics 27 software. The *p* < 0.05 significance level indicates that there is a significant difference between the values. Figures were plotted with GraphPad Prism(version 9.0.0).

## 3. Results

### 3.1. Antibacterial Activity of the Antimicrobial Peptide Cyclo-zp80r

The MIC of cyclo-zp80r against *Salmonella* strains ranged from 2 to 8 μM. Its MIC against *L. monocytogenes* ranged from 4 to 8 μM. This indicates that cyclo-zp80r is effective against both Gram-negative and Gram-positive bacteria.

### 3.2. Effects of Cyclo-zp80r on the Growth and Survival of S. enterica and L. monocytogenes

**Figure 1:**
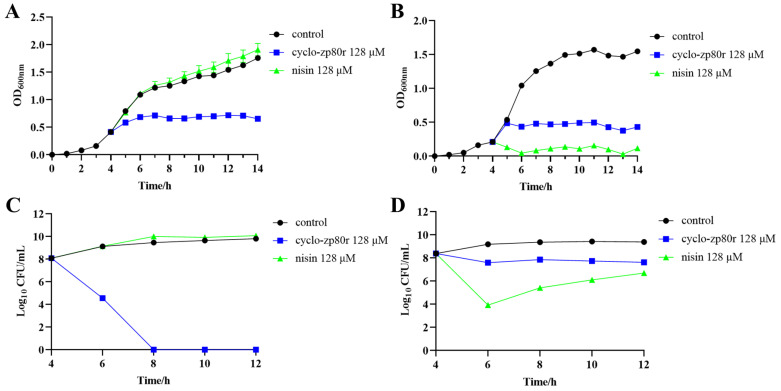
Growth and time–kill curves of cyclo-zp80r against *S. enterica* and *L. monocytogenes.* (**A**) The effect of cyclo-zp80r on the growth curve of *S. enterica*; (**B**) the effect of cyclo-zp80r on the growth curve of *L. monocytogenes*; (**C**) the effect of cyclo-zp80r on the time–kill curve of *S. enterica*; (**D**) the effect of cyclo-zp80r on the time–kill curve of *L. monocytogenes*.

To better understand the effect of cyclo-zp80r on *S. enterica* and *L. monocytogenes*, the growth and time–kill curves of both strains were generated. The growth curves (Figure 1A,B) showed that the negative control group grew rapidly, and *L. monocytogenes* reached a stable phase after 6–8 h. Treatment with 128 μM cyclo-zp80r significantly inhibited the growth of both pathogens, resulting in significantly lower final cell densities compared to the negative control group. In addition, the inhibition of *S. enterica* by 128 μM cyclo-zp80r treatment (Figure 1A) was significantly greater than that of the bio-preservative control (128 μM nisin). The time–kill curves (Figure 1C,D) show the survival of *S. enterica* and *L. monocytogenes* after cyclo-zp80r treatment. According to the time–kill curve for *S. enterica* (Figure 1C), the number of viable cells decreased to an undetectable level after 4 h of treatment with 128 μM cyclo-zp80r. The bio-preservative control group had no inhibitory effect at all. However, the lethal effect of 128 μM cyclo-zp80r on *L. monocytogenes* (Figure 1D) was not obvious, with an almost stable viable count. The 128 μM nisin exhibited a better lethal effect in the first 2 h, but the number of viable *L. monocytogenes* began to increase after 2 h. This indicates that 128 μM cyclo-zp80r has a bactericidal effect on *S. enterica* and a bacteriostatic effect on *L. monocytogenes.*

### 3.3. The Antibacterial Effect of Cyclo-zp80r Against S. enterica and L. monocytogenes in NFC Orange Juice

**Figure 2:**
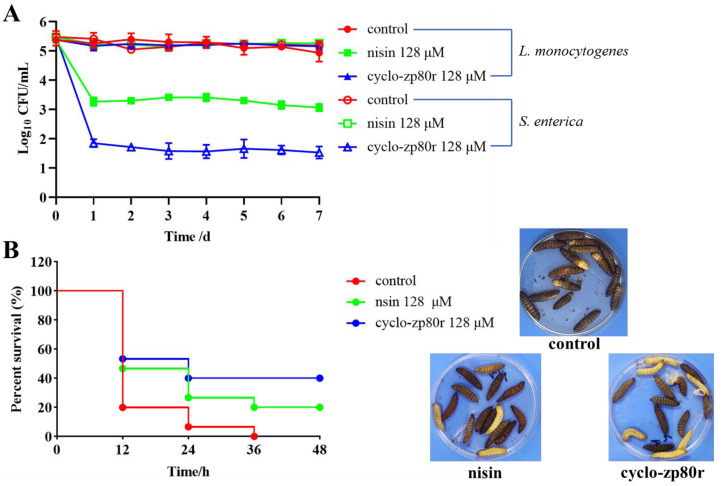
The antibacterial effect of cyclo-zp80r on *S. enterica* and *L. monocytogenes* in NFC orange juice. (**A**) Viable counts of *S. enterica* and *L. monocytogenes* in NFC orange juice; (**B**) detoxification effect of cyclo-zp80r on *G. mellonella* exposed to *S. enterica* and *L. monocytogenes* in NFC orange juice. The control was NFC orange juice contaminated with *S. enterica* and *L. monocytogenes* without peptide treatment; the plates show the phenotype of *G. mellonella* larvae.

As shown in Figure 2A, the viable numbers of *S. enterica* and *L. monocytogenes* in NFC orange juice were measured over 7 consecutive days and expressed as Log_10_ CFU/mL. The numbers of viable *S. enterica* and *L. monocytogenes* in the negative control group remained stable at 4.9–5.5 Log_10_ CFU/mL. *L. monocytogenes* in the negative control group did not grow, which may be due to the low pH and the presence of antibacterial phenolic compounds in the orange juice [36,37]. The bio-preservative control (128 μM nisin) effectively reduced *L. monocytogenes*, with its count dropping to 3.1–3.4 Log_10_ CFU/mL after 1 d. The 128 μM cyclo-zp80r treatment had a strong killing effect on *S. enterica*, with the number of viable *S. enterica* decreasing to 1.5–1.9 Log_10_ CFU/mL after 1 d. Over the subsequent six days, the lethality rate of *S. enterica* in the cyclo-zp80r treatment group remained at 99.99%. The results indicate that cyclo-zp80r can effectively reduce *S. enterica* in NFC orange juice and maintain a low level of contamination.

Figure 2B shows the detoxification effect of cyclo-zp80r on *G. mellonella* exposed to *S. enterica* and *L. monocytogenes* in NFC orange juice. The negative control group had high levels of *S. enterica* (5.0–5.3 Log_10_ CFU/mL) and *L. monocytogenes* (4.6–5.2 Log_10_ CFU/mL), resulting in 0% survival rate of *G. mellonella* after 36 h. The bio-preservative control group (128 μM nisin) had a high level of *S. enterica* (5.2–5.3 Log_10_ CFU/mL) and a moderate level of *L. monocytogenes* (3.0–3.2 Log_10_ CFU/mL), with a 20% survival rate of *G. mellonella* at 48 h. The cyclo-zp80r treatment group had a low level of *S. enterica* (1.3–1.7 Log_10_ CFU/mL) and a high level of *L. monocytogenes* (5.1–5.2 Log_10_ CFU/mL), resulting in the survival rate of *G. mellonella* increasing to 40% at 48 h.

### 3.4. The Effect of Cyclo-zp80r on Microbes in NFC Orange Juice

**Figure 3:**
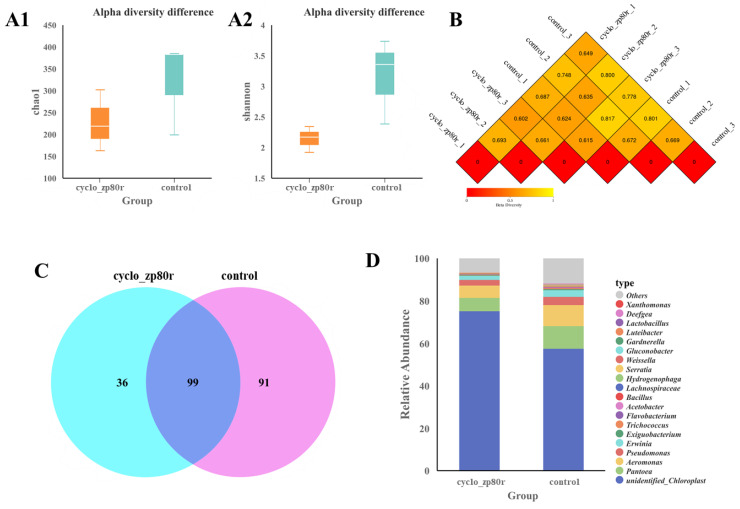
Microbiome analysis of NFC orange juice after cyclo-zp80r treatment. (**A**) Analysis of alpha diversity differences based on Chao1 index (**A1**) and Shannon index (**A2**); (**B**) beta diversity based on unweighted unifrac; (**C**) Venn diagram of each group at the genus classification level; (**D**) histogram of the relative abundance of each group at the genus classification level.

Figure 3A shows the alpha diversity differences between the control group and the cyclo-zp80r treatment group. The chao1 index (Figure 3(A1)) indicates lower species abundance in the cyclo-zp80r treatment group. The Shannon index (Figure 3(A2)) shows reductions in both species abundance and species evenness after cyclo-zp80r treatment. Beta diversity, based on unweighted unifrac, reveals species presence/absence information. A higher value indicates a greater difference between samples. As shown in Figure 3B, values range from 0.602 to 0.817, suggesting significant species differences between samples. A Venn diagram (Figure 3C) was used to evaluate the differences between the control and cyclo-zp80r treatment groups at the genus level. The results showed that there were 99 overlapping genera between the two groups. The cyclo-zp80r group had a smaller number of unique genera. The top 20 species with the highest relative abundances at the genus level were detected in the orange juice of the two groups (Figure 3D). Among bacteria, *Pantoea* accounted for the largest proportion, followed by *Aeromonas*, *Pseudomonas*, and *Erwinia*. *Pantoea* is an endophyte of citrus [38] and is prevalent in the vector of citrus Huanglongbing [39]. *Aeromonas* and *Pseudomonas* are spoilage-causing agents in various food products [40]. *Erwinia* causes rot of plant tissue by producing cell-wall-degrading enzymes [41]. Cyclo-zp80r treatment induced a decrease in relative abundance from 10.60% to 6.35%, 9.95% to 5.80%, 3.95% to 2.51%, and 0.14% to 0.08% for *Pantoea*, *Aeromonas*, *Pseudomonas*, and *Erwinia*, respectively. This indicates that cyclo-zp80r can decrease the diversity and abundance of bacteria in orange.

### 3.5. The Stability of Cyclo-zp80r

The thermal stability and enzymatic stability of cyclo-zp80r in orange juice were determined to evaluate its application potential in NFC orange juice processing. The results are shown in Table 1. After treatment at high temperature (121 °C, 5 min) and pasteurization (65 °C, 30 min), as well as incubation of fresh orange juice at 37 °C for 2 h, the MIC of cyclo-zp80r against *S. enterica* remained at 4 μM. This indicates that the antimicrobial peptide cyclo-zp80r has good stability. Industrial high-temperature sterilization or pasteurization processes do not diminish its inhibitory activity against *S. enterica*.

### 3.6. Membrane Depolarization by Cyclo-zp80r

**Figure 4:**
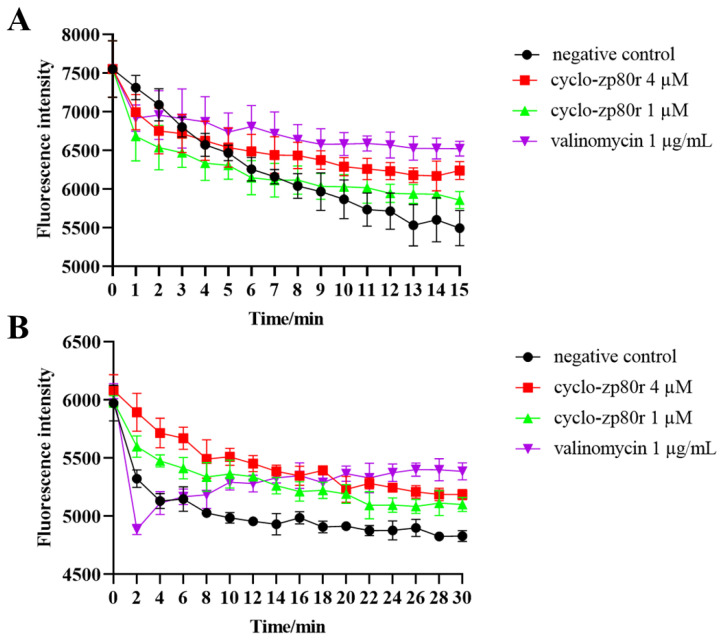
The effect of cyclo-zp80r on cell membrane potential. (**A**) The effect of cyclo-zp80r on the membrane potential of *S. enterica*; (**B**) the effect of cyclo-zp80r on the transmembrane potential of *L. monocytogenes*.

Membrane potential is a source of free energy. It plays an important role in bacterial antibiotic resistance, pH homeostasis, membrane transport, cell division, and environmental sensing [42]. DiSC_3_(5) is a membrane-potential-sensitive probe. When the membrane is depolarized, the fluorescence intensity increases [43]. Fluorescence enhancement is positively correlated with the degree of membrane potential loss. As shown in Figure 4, the fluorescence intensity of the negative control group decreased rapidly. The fluorescence quenching was disturbed by the positive control (1 μg/mL valinomycin). The fluorescence intensities of 1 and 4 μM cyclo-zp80r treatments were higher than those of the negative control, and slightly lower than those of the valinomycin treatment. Similar trends in change were observed in both *S. enterica* and *L. monocytogenes*. The results indicate that cyclo-zp80r had a depolarization effect on *S. enterica* and *L. monocytogenes.*

### 3.7. Effect of Cyclo-zp80r on the Outer Membrane of S. enterica and Observation of the Ultrastructure

**Figure 5:**
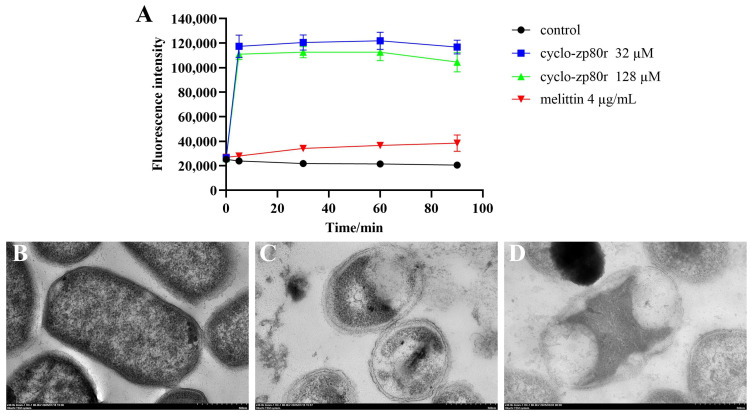
The effect of cyclo-zp80r on the permeability of the outer membrane and cell structure of *S. enterica*. (**A**) Permeability of the outer membrane of *S. enterica*; (**B**) TEM images of *S. enterica* treated with 0 μM cyclo-zp80r; (**C**) TEM images of *S. enterica* treated with 32 μM cyclo-zp80r; (**D**) TEM images of *S. enterica* treated with 128 μM cyclo-zp80r.

To further determine the effect of cyclo-zp80r on the membrane, the study focused on the outer membrane of *S. enterica*. Firstly, the effect of cyclo-zp80r on the permeability of the outer membrane of *S. enterica* was investigated using NPN (Figure 5A). The negative control had stable fluorescence intensities. Melittin is a typical pore-formation peptide, and 4 μg/mL of it induced an increase in fluorescence intensity. Surprisingly, significant enhancement of fluorescence intensity was observed following treatment with 32 μM and 128 μM cyclo-zp80r. This indicates that cyclo-zp80r has a destructive effect on the outer membrane of *S. enterica*. The ultrastructure of *S. enterica* was also observed using TEM. The control group had smooth and complete cell outlines (Figure 5B). Their cytoplasmic membranes closely adhered to the cell wall, and the cytoplasm was evenly distributed. In contrast, 32 μM cyclo-zp80r treatment induced the separation of the cytoplasm from the cell wall (Figure 5C). Their outer membranes became rough and fractured, with uneven distribution of cytoplasmic content. The separation of the cytoplasm from the cell wall and the uneven distribution of cytoplasmic content intensified when the concentration of cyclo-zp80r increased to 128 μM (Figure 5D). The rupture of the cell membrane resulted in an outflow of cytoplasmic content and showed cavitation. The results indicate that cyclo-zp80r can damage the cell membrane structure.

### 3.8. Effect of Cyclo-zp80r on the Cell Morphology of S. enterica and L. monocytogenes

**Figure 6:**
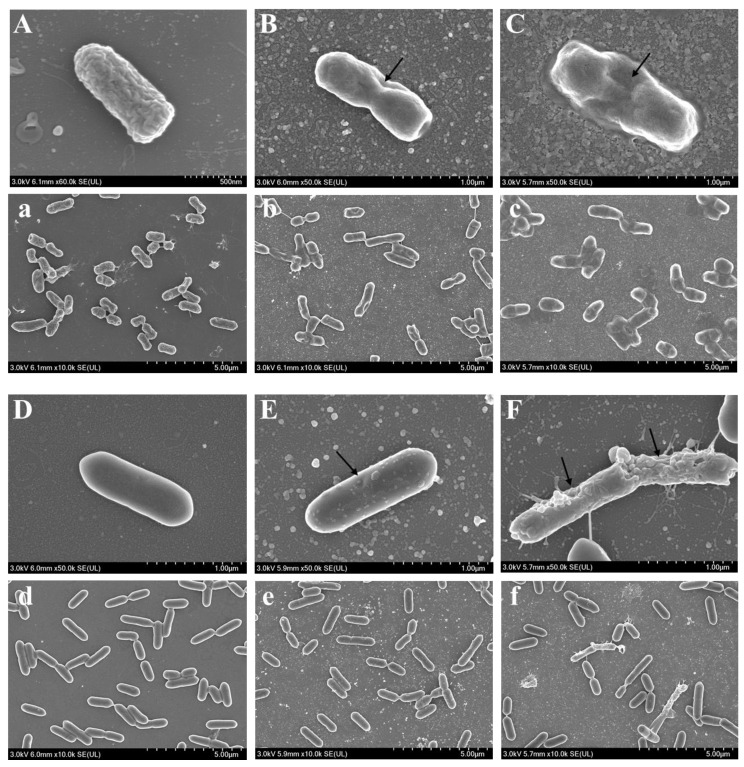
Scanning electron microscopy images of *S. enterica* and *L. monocytogenes* following cyclo-zp80r treatment. (**A**,**a**) *S. enterica* treated with 0 μM cyclo-zp80r; (**B**,**b**) *S. enterica* treated with 32 μM cyclo-zp80r; (**C**,**c**) *S. enterica* treated with 128 μM cyclo-zp80r; (**D**,**d**) *L. monocytogenes* treated with 0 μM cyclo-zp80r; (**E**,**e**) *L. monocytogenes* treated with 32 μM cyclo-zp80r; (**F**,**f**) *L. monocytogenes* treated with 128 μM cyclo-zp80r.

For *S. enterica*, the cells in the control group were full and intact (Figure 6A,a); however, significant changes in cell morphology were observed after cyclo-zp80r treatment. At 32 μM cyclo-zp80r, the cells inwardly collapsed (Figure 6B,b), and this collapse became more pronounced at 128 μM cyclo-zp80r (Figure 6C,c). This collapse may result from the leakage of cytoplasmic content due to membrane damage. For *L. monocytogenes*, the cells in the control group were full, with smooth and intact surfaces (Figure 6D,d). The cells remained full after treatment with 32 μM cyclo-zp80r, but their surfaces became rough with granular protrusions (Figure 6E,e). The deformation of the cell surface significantly intensified after treatment with 128 μM cyclo-zp80r. The cell surfaces showed localized indentations, distortions, and bubble-like protrusions (Figure 6F,f). Holes formed as the concentration of cyclo-zp80r increased (black arrows in Figure 6E,F). The size of the holes was positively correlated with treatment concentration.

### 3.9. Effect of Cyclo-zp80r on Reactive Oxygen Species Production in S. enterica and L. monocytogenes

**Figure 7:**
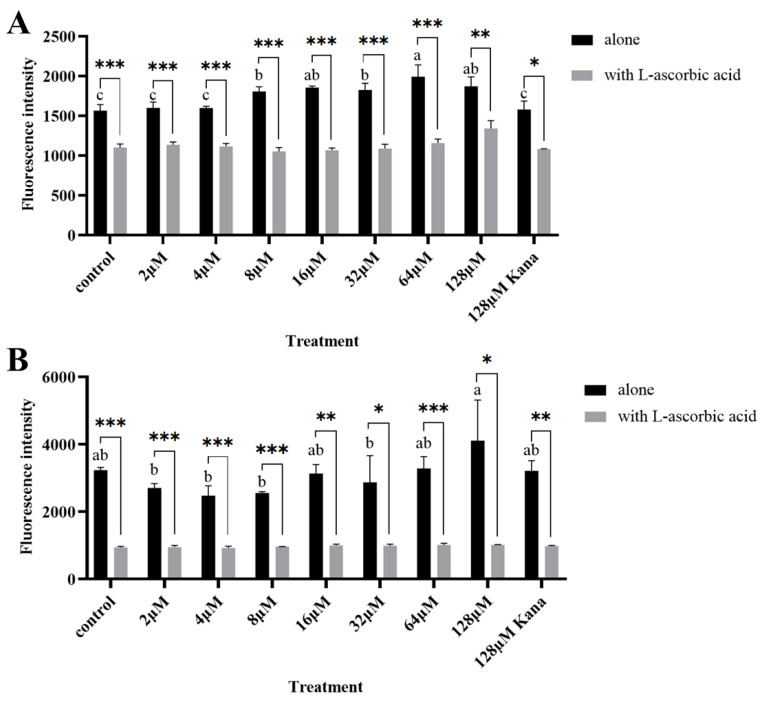
The effect of cyclo-zp80r on reactive oxygen species production in *S. enterica* (**A**) and *L. monocytogenes* (**B**). * indicates signiffcantly different at the 0.05 level, ** indicates signiffcantly different at the 0.01 level, *** indicates signiffcantly different at the 0.001 level. Different letters indicate signiffcantly different at the 0.05 level.

High levels of reactive oxygen species (ROS) can cause serious damage to cellular proteins and DNA and disrupt bacterial cell homeostasis [44,45]. To investigate the effect of cyclo-zp80r on the production of ROS in *S. enterica* and *L. monocytogenes*, the level of ROS was determined using the DCFH-DA fluorescence method. The results are shown in Figure 7. For both *S. enterica* (Figure 7A) and *L. monocytogenes* (Figure 7B), ROS levels in the alone group were significantly higher than those in the L-ascorbic acid group, indicating the production of ROS in both pathogens. In *S. enterica*, the ROS content increased with cyclo-zp80r concentration, reaching a maximum of 64 μM. Kanamycin has been reported to induce ROS production [46]. Cyclo-zp80r induced the production of significantly higher ROS contents than 128 μM kanamycin at concentrations from 8 μM to 128 μM. For *L. monocytogenes*, there was no significant difference between the cyclo-zp80r treatment group and the control group; however, 128 μM cyclo-zp80r induced the production of high ROS in *L. monocytogenes*. The results indicate that cyclo-zp80r can induce ROS production, particularly in *S. enterica*.

### 3.10. The Effect of Cyclo-zp80r on DNA

**Figure 8:**
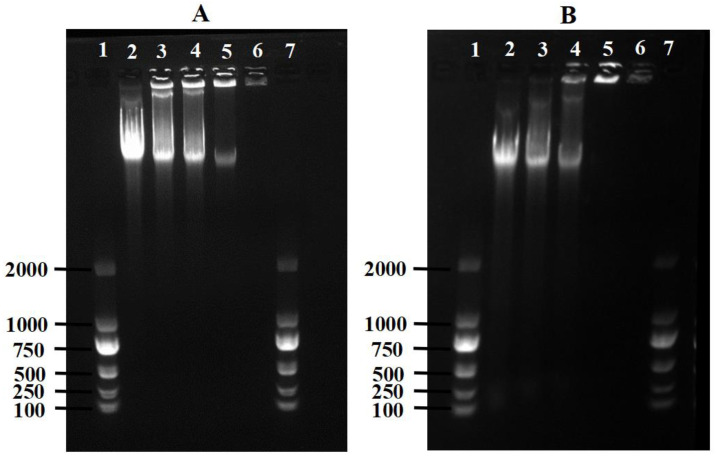
Binding of *S. enterica* (**A**) and *L. monocytogenes* (**B**) DNA with cyclo-zp80r. Lanes 1 and 7 contained the DNA marker DL2000; lanes 2–6 contained DNA/cyclo-zp80r at concentrations of 1:0, 1:0.125, 1:0.25, 1:0.5, 1:1, respectively.

Gel retardation assays can illustrate the binding between cyclo-zp80r and DNA. When bound, DNA movement in agarose gel is delayed. Binding between cyclo-zp80r and *S. enterica* DNA was observed within the tested concentration ratios of 1:0.125 to 1:1, as shown in Figure 8A. The extent of DNA binding increased with the cyclo-zp80r concentration. At a concentration ratio of 1:1, *S. enterica* DNA fully bound to cyclo-zp80r, halting DNA movement. For *L. monocytogenes*, DNA completely bound to cyclo-zp80r once the concentration ratio reached 1:0.5. The results indicate that there is a binding interaction between cyclo-zp80r and the DNA of both *S. enterica* and *L. monocytogenes*.

## 4. Discussion

NFC orange juice satisfies consumer demand for nutrients and flavor; however, it faces the challenge of residual microbes. To prevent microbial growth, NFC orange juice is usually stored at 0–6 °C. *S. enterica* and *L. monocytogenes* are two important foodborne pathogens that can survive under low temperatures in NFC orange juice following contamination. The two pathogens pose a threat to the safety of NFC orange juice and require special attention. In this study, we investigated the application potential of cyclo-zp80r in controlling *S. enterica* and *L. monocytogenes* in NFC orange juice. Cyclo-zp80r exhibited excellent thermal stability (Table 1), even at 121 °C for 5 min. This indicates that cyclo-zp80r can withstand the pasteurization of NFC orange juice. Our previous study showed that it has good protease stability [24]. In the current study, we showed that cyclo-zp80r can resist the enzymes present in fresh orange juice (Table 1); therefore, it can be added to the juice before and after pasteurization. From a processing perspective, cyclo-zp80r can be applied to NFC orange juice.

The antimicrobial potential of cyclo-zp80r was systematically investigated. It exhibited MIC values ranging from 2 μM (0.0056 mg/mL) to 8 μM (0.0222 mg/mL) against *S. enterica* and *L. monocytogenes*. To date, numerous antimicrobial compounds have been studied to control the two pathogens. The MIC values of *Lactobacillus curvatus* B67-derived postbiotic against *S. enterica* and *L. monocytogenes* were 40 mg/mL and 78 mg/mL, respectively. The MIC values of quercetin against *S. enterica* and *L. monocytogenes* were 0.2 mg/mL and 0.3 mg/mL, respectively [47]. The MIC value of phenolic extracts from *Ruta graveolens* against *L. monocytogenes* was 1.25 mg/mL [48]. The MIC values of antibacterial peptides (ε-PL) against *Salmonella* Enteritidis and *L. monocytogenes* were 0.031–1.0 mg/mL [49]. Cyclo-zp80r was much more effective than these antimicrobial compounds. Nisin is the most widely used food bio-preservative [50] and was used as a control in this study. Based on the growth curves (Figure 1A,B), 128 μM cyclo-zp80r inhibited the growth of both *S. enterica* and *L. monocytogenes*, while 128 μM nisin only inhibited the growth of *L. monocytogenes*. Based on the time–kill curves (Figure 1C,D), 128 μM cyclo-zp80r treatment resulted in the death of 100% of *S. enterica* and 83.70% of *L. monocytogenes*, while 128 μM nisin treatment only resulted in the death of 99.99% of *L. monocytogenes*. The antibacterial effectiveness of cyclo-zp80r in NFC orange juice was also evaluated. Cyclo-zp80r exhibited strong antibacterial effectiveness against *S. enterica*, with a reduction in viable count of 3.6 Log_10_ CFU/mL after 1 d of treatment (Figure 2A). Nisin exhibited strong antibacterial effectiveness against *L. monocytogenes*, with a reduction in viable count of 2.1 Log_10_ CFU/mL after 1 d of treatment (Figure 2A). The results indicate that cyclo-zp80r has excellent antibacterial activity, particularly against *S. enterica*. *S. enterica* and *L. monocytogenes* are pathogenic at high concentrations [51,52]. After 7 days of storage at 4 °C, the NFC orange juice in the control group resulted in the death of 100% of *G. mellonella* due to high concentrations of *S. enterica* and *L. monocytogenes* (Figure 2B). However, the death rate after nisin treatment was 80% due to reduced concentrations of *L. monocytogenes*. The death rate after cyclo-zp80r treatment reduced to 60% due to reduced concentrations of *S. enterica*. The results indicate that 128 μM cyclo-zp80r treatment was better at controlling *S. enterica* and *L. monocytogenes* in NFC orange juice than 128 μM nisin treatment. In addition, microbiome analysis revealed a reduction in species diversity and abundance in NFC orange juice following cyclo-zp80r treatment (Figure 3C,D). Dominant genera, including *Pantoea*, *Aeromonas*, *Pseudomonas*, and *Erwinia*, which are common food spoilage bacteria [53,54,55], were significantly decreased after the treatment; therefore, cyclo-zp80r has great potential in ensuring the safety of NFC orange juice. Cyclo-zp80r treatment may help extend shelf life.

Cyclo-zp80r exhibited close MIC values against *S. enterica* and *L. monocytogenes*, but had significantly different lethal effects on the two pathogens. A 99.9% reduction is the threshold that distinguishes bactericidal action from bacteriostatic action [23]. Cyclo-zp80r exhibited bactericidal action against *S. enterica* while showing bacteriostatic action against *L. monocytogenes*. Understanding the mechanism of action can reveal the differences in the mode of action. Unlike antibiotics, antimicrobial peptides employ multiple antibacterial mechanisms to inhibit or kill harmful bacteria in different ways [56]. These mechanisms include destroying the cell membrane, interfering with the synthesis of nucleic acids (DNA, RNA) and proteins, hindering cell wall synthesis, and inducing ROS production [56,57]. Membrane potential can indicate the impact of antimicrobial compounds on the cell membrane [58]. To explore the effect of cyclo-zp80r on the cell membrane, the fluorescence probe DiSC_3_ (5) was used to determine the change in cell membrane potential after cyclo-zp80r treatment. The results (Figure 4) showed that the cell membrane was depolarized, suggesting membrane damage. *S. enterica* is a Gram-negative bacterium, while *L. monocytogenes* is a Gram-positive bacterium. The primary difference in their membrane structure is that *S. enterica* has an outer membrane. The impact of cyclo-zp80r on this outer membrane was further investigated. The results of the analysis of outer membrane permeability tests (Figure 5A) indicated that cyclo-zp80r has a strong destructive effect on the outer membrane of *S. enterica*. TEM was used to visualize this disruptive effect on the outer membrane (Figure 5B), which resulted in deformation and rupture. Large empty areas were observed within the cytoplasm due to the leakage of cytoplasmic substances. This also indicates damage to the outer membrane. Furthermore, the effect of cyclo-zp80r on the morphologies of both *S. enterica* and *L. monocytogenes* cells was observed using SEM (Figure 6). Cyclo-zp80r caused distinct changes, with collapse being the primary feature for *S. enterica* and localized bubble-like protrusions being the primary feature for *L. monocytogenes*. Based on the cell plumpness, cytoplasmic leakage was more severe in *S. enterica*. We deduce that membrane structure is the primary target of cyclo-zp80r.

To further understand possible intracellular targets of cyclo-zp80r, ROS production was investigated. The results showed that cyclo-zp80r treatment induced ROS production in *S. enterica* in a concentration-dependent manner (Figure 7A). However, the induction of ROS production in *L. monocytogenes* was not significant (Figure 7B). Vila Domínguez et al.’s research [59] also showed differences in the induction of ROS production between Gram-positive and Gram-negative bacteria. This difference may be due to the varying amounts of antimicrobial compounds entering the cell, depending on the extent of damage to the cell envelope. In addition, the result of the gel retardation assay (Figure 8) shows that cyclo-zp80r can bind strongly to the DNA of *S. enterica* and *L. monocytogenes*. Zp80, the prodrug of cyclo-zp80r, bound to *L. monocytogenes* DNA at a concentration ratio (peptide: DNA) ≥ 0.0625:1 [23]. Cyclo-zp80r retains the DNA-binding activity of zp80. Therefore, the antibacterial mechanism of cyclo-zp80r may involve (1) targeting the cell membrane, disrupting its structure and causing cytoplasmic leakage; (2) targeting intracellular DNA, disturbing its normal function; and (3) inducing ROS production and interfering with cell metabolism and structure.

## 5. Conclusions

In summary, the cyclic peptide cyclo-zp80r has antimicrobial activity against both *S. enterica* and *L. monocytogenes*. The pathogenicity of *S. enterica* and *L. monocytogenes* in NFC orange juice was reduced after cyclo-zp80r treatment. The abundance of spoilage bacteria in NFC orange juice was also reduced. Cyclo-zp80r controlled *S. enterica* and *L. monocytogenes* via multiple mechanisms, including damaging the cell membrane, binding to DNA, and inducing ROS production. This shows significant potential for ensuring the safety of NFC orange juice. This study broadened the potential application of cyclo-zp80r from the pharmaceutical industry to the food industry. Cyclo-zp80r exhibited excellent stability, which contributes to its application in the food industry. Future studies will focus on the interaction between cyclo-zp80r and food components.

## Figures and Tables

**Table 1:** Stability of cyclo-zp80r.

Treatment Condition	High Temperature (121 °C, 5 min)	Pasteurization (65 °C, 30 min)	Fresh Orange Juice (37 °C, 2 h)
MIC value (μM)	4	4	4

## Data Availability

The original contributions presented in this study are included in the article. Further inquiries can be directed to the corresponding author.
